# Development and validation of a pharmacokinetic tool to convert random topiramate concentrations to standardized trough levels in bipolar disorder

**DOI:** 10.3389/fphar.2026.1867058

**Published:** 2026-09-08

**Authors:** Xuan Jv, Shuzhen Li, Ye Liu, Qianqian Cao, Gaopeng Li, Mingxia Chen, Jipeng Liu, Jiezheng Dong

**Affiliations:** 1 Department of Psychiatry, The Seventh People’s Hospital of Hangzhou (Affiliated Mental Health Center, Zhejiang University School of Medicine), Hangzhou, Zhejiang Province, China; 2 Qilihe District People’s Hospital, Lanzhou, Gansu Province, China; 3 Gansu Provincial Hospital of Traditional Chinese Medicine, Lanzhou, Gansu Province, China; 4 Xiaoshan Third People’s Hospital, Hangzhou, Zhejiang Province, China; 5 Jiashan County First People’s Hospital, Jiaxing, Zhejiang Province, China; 6 Affiliated Hospital of Shaoxing University of Arts and Sciences, Shaoxing, Zhejiang Province, China; 7 Fourth Clinical Medical College of Zhejiang University of Traditional Chinese Medicine, Hangzhou, Zhejiang Province, China

**Keywords:** bipolar disorder, concentration conversion, mood stabilizer, pharmacokinetics, therapeutic drug monitoring, topiramate, trough concentration

## Abstract

**Background:**

Topiramate is sometimes used in patients with bipolar disorder (BD), though off-label, warranting therapeutic drug monitoring (TDM) for safety. Guidelines recommend trough-level sampling (12–16h post-dose), but random levels are common, complicating interpretation. A tool to convert random topiramate concentrations to a standardized trough equivalent is lacking.

**Objective:**

To develop and validate a pharmacokinetic (PK) tool for estimating the standardized trough concentration at 14h post-dose (C_14_) from a random sample in patients receiving topiramate.

**Methods:**

A conversion model was derived using a one-compartment model with population PK parameters for immediate-release topiramate (elimination half-life = 25h, time-to-peak = 3h). A conversion coefficient (Kt) table was generated. The tool was validated against 183 paired TDM samples from inpatients with BD on stable topiramate monotherapy, using the measured C_14_ as reference. Reliability, agreement, and accuracy were assessed *via* intraclass correlation coefficient (ICC), Bland-Altman analysis, and mean absolute percentage error (MAPE).

**Results:**

The tool demonstrated excellent reliability (ICC = 0.92, 95% CI: 0.90–0.94) and agreement (mean bias = +0.008 μg/mL). Predictive accuracy was high (MAPE = 6.3%), with 97.3% of estimates having an absolute error <15%. Performance was consistent across pharmacokinetic phases and subgroups (age, dose).

**Conclusion:**

This preliminary PK conversion tool reliably estimates the standardized trough concentration of immediate-release topiramate from random TDM levels. It could potentially aid safety monitoring by improving the interpretation of non-standard samples, though its clinical utility requires prospective external validation and linkage to patient outcomes.

## 1 Introduction

Topiramate is usually used in clinical practice for patients with bipolar disorder (BD), though primarily as an off-label adjunctive agent rather than a first-line mood stabilizer. While not recommended as a primary treatment in most international guidelines due to limited efficacy evidence from large-scale randomized controlled trials for core mood episodes (Vieta et al., 2018), it continues to be considered in specific contexts, such as for weight management or in patients with comorbid conditions (e.g., migraine, epilepsy) when first-line agents are not suitable or tolerated (Sills et al., 2020). Its core mechanism of action, which involves inhibition of voltage-gated sodium and calcium channels, enhancement of γ-aminobutyric acid (GABA)ergic neurotransmission, and antagonism of α-amino-3-hydroxy-5-methyl-4-isoxazolepropionic acid (AMPA)/kainate receptors, contributes to its anticonvulsant activity, neuroprotective properties, and potential mood-modulating effects (Yatham et al., 2018). In these off-label clinical settings, therapeutic drug monitoring (TDM) is crucial to optimize safety and minimize dose-related adverse effects.

Topiramate exhibits significant inter-individual variability in pharmacokinetics, making TDM essential for optimizing exposure relative to individual tolerance. For the immediate-release topiramate formulation investigated, consensus guidelines from the Arbeitsgemeinschaft für Neuropsychopharmakologie und Pharmakopsychiatrie (AGNP) and the International Association of Therapeutic Drug Monitoring and Clinical Toxicology (IATDMCT) recommend steady-state trough concentration (12–16 h post-dose) as the standard (Hiemke et al., 2011; Pennazio et al., (2022)). The 12–16 h window, with C14 (14 h post-dose) as the average standard, was selected as the standardized trough in this study, aligning with guideline recommendations and clinical practice (e.g., evening dosing with morning sampling). The C14 time point represents the midpoint of this window, providing a single, clinically practical target for estimation.

Topiramate is widely prescribed and it has an oral bioavailability of 80%–95%, with minimal food effect. For the immediate-release topiramate formulation investigated in this study, pharmacokinetic studies report a time to peak plasma concentration (Tmax) at approximately 3 h post-dose (range 2–4 h) and an elimination half-life (t1/2) averaging 25 h (range 20–30 h) (Doose et al., 1996; Bourgeois et al., 1999). Topiramate is primarily eliminated unchanged in the urine (70%–80%), with a smaller fraction metabolized in the liver via hydroxylation and glucuronidation (Britzi et al., 2005). Factors such as renal function, age, body weight, and concurrent medications (e.g., carbonic anhydrase inhibitors, phenytoin, carbamazepine) can significantly alter its clearance, further emphasizing the need for rigorous TDM (Patsalos et al., 2008).

Despite clear guidelines, adherence to the 12–16 h post-dose sampling window is often logistically challenging in inpatient and outpatient settings. Blood samples are frequently collected at random times (e.g., routine morning rounds, emergency visits, patient non-compliance), leading to non-standard TDM samples (Schoretsanitis et al., 2018). Although the elimination half-life of topiramate formulation results in a relatively stable concentration-time profile, significant variations in plasma drug concentrations still exist at different time points within a 24-h period in vivo (Contin et al., 2016). Direct comparison of random concentrations to trough-based therapeutic ranges may lead to inappropriate dose adjustments—risks that are particularly significant given significant inter-individual variability necessitating TDM (Jann et al., 2018).

Currently, no widely accepted, validated tool exists to correct random topiramate concentrations to a standardized trough equivalent. Previous small-scale studies have attempted to address this gap but lacked large-scale validation, or comprehensive sampling time coverage (Hsu et al., 2024). This study aimed to develop, validate, and provide a preliminary pharmacokinetic-based tool to convert random plasma topiramate concentrations to estimated steady-state trough concentrations (C14), the average standard within the 12–16 h post-dose sampling window. We derived simplified conversion formulas, generated a comprehensive conversion coefficient table, and validated the system using real-world TDM data from 183 inpatients. The goal is to enhance the interpretability of non-standard TDM samples and support personalized pharmacotherapy for individuals with BD.

## 2 Experimental procedures

### 2.1 Pharmacokinetic parameter synthesis

Core population pharmacokinetic (PK) parameters for immediate-release topiramate were synthesized from authoritative literature, population PK analyses, and product information (Mould and Upton, 2012). Parameters were selected for consistency across studies and relevance to steady-state modeling:

- Elimination half-life (t_1_/_2_): 25 h (population mean, range 20–30 h, based on formulation-specific clinical data).
- Elimination Rate Constant (k): Calculated as k = ln2/t_1_/_2_ ≈ 0.693/25 ≈ 0.0277 h^-1^ (derived from t_1_/_2_ = 25 h, Refs ^[7,8]^).
- Time to Peak Concentration (Tmax): 3 h (population mean, range 2–4 h, based on formulation-specific clinical data, used to demarcate the absorption/distribution phase from the post-absorption phase).

(Note: For a Immediate-Release Formulation with a Tmax of ∼3 h, the classical ‘absorption rate constant (k_a_)” derived from an immediate-release model is not applicable. The prolonged absorption process is better characterized by the time to peak.

Standard Trough Sampling Window: 12–16 h post-dose, with C_14_ (14 h post-dose) as the average standard, consistent with AGNP/IATDMCT guidelines.)

### 2.2 Model development and formula derivation

#### 2.2.1 Core assumptions

The conversion model was based on three standard PK modeling assumptions (Gabrielsson and Weiner, 2016):

- Patients are at pharmacokinetic steady state (≥5 half-lives, ≥125 h of stable dosing).
- Topiramate’s PK is adequately described by a one-compartment open model with first-order absorption and elimination.
- Population-average PK parameters are used, with inter-individual variability evaluated as residual error during validation.

#### 2.2.2 Simplified formula derivation

Absorption/Distribution Phase (1 ≤ t ≤ 3 h post-dose).

To aid non-specialist readers, we first provide a conceptual summary. For immediate-release topiramate, the time to peak (Tmax) is about 3 h, meaning that absorption is largely complete by that time. Because the elimination half-life is long (25 h), the concentration declines slowly after the peak. The conversion coefficient Kt = C_14_/Ct is therefore larger than one for very early samples (e.g., 1 h) and falls below one near the peak, reflecting the shape of the concentration–time curve. The mathematical derivation below formalizes this logic.

During this phase, topiramate is being absorbed and plasma concentrations rise toward the peak. To estimate the standardized trough concentration at 14 h (C_14_) from an early random sample, conversion coefficients (Kt = C_14_/Ct) were derived using a one-compartment oral absorption model under steady-state conditions (dosing interval τ = 24 h), with first-order absorption and elimination.

The absorption rate constant (ka) was determined from the reported Tmax of approximately 3 h (Garnett et al., 2021; Doose et al., 1996) using the standard one-compartment Tmax formula (Gabrielsson and Weiner, 2016): Tmax = ln (ka/k)/(ka–k). With k = 0.0277 h^-1^ and Tmax = 3 h, iterative solving gives ka ≈0.5 h^-1^.

The steady-state concentration at time t after a dose is proportional to:

$$C\left(t\right)\propto 1‐e‐k\tau e‐\text{kt}‐e‐\text{kat}$$

**FIGURE 1:**
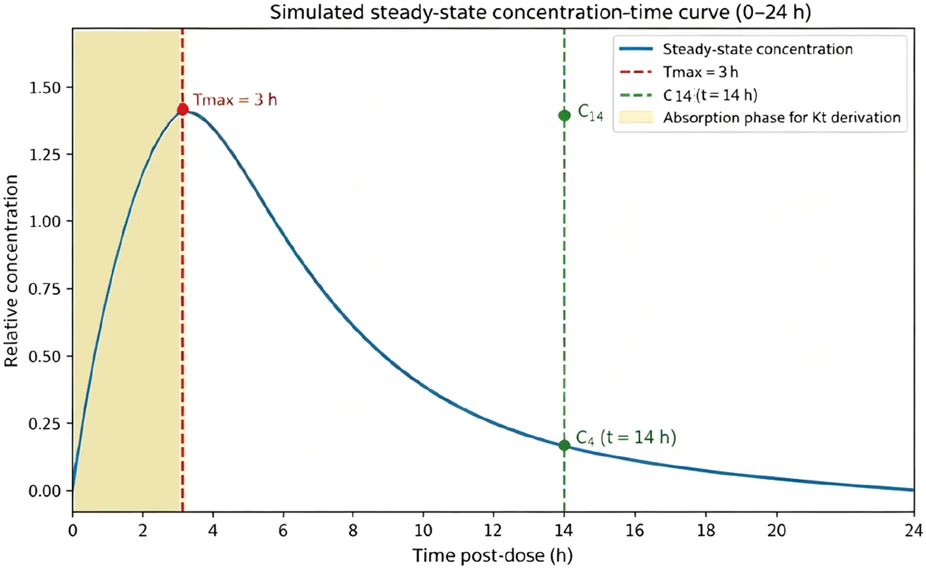
Simulated steady-state concentration-time profile (0–24 h) illustrating the pharmacokinetic basis for the conversion tool.

Because Kt = C_14_/Ct, the dose, bioavailability, and volume of distribution cancel out, allowing calculation solely from the rate constants. The concentration-time profile was simulated numerically in Python 3.9 for t = 0–24 h. The resulting Kt values at t = 1, 2, and 3 h are 1.852, 1.171, and 0.971, respectively. At Tmax (t = 3 h), Kt = 0.971 < 1, confirming that C_14_ is lower than the peak concentration, as expected physiologically. A plot of the simulated profile is provided in Figure 1 (Supporting Information).

Post-Absorption (Elimination) Phase (t > 3 h post-dose).

After 3 h, absorption is essentially complete. Plasma concentrations decline mono-exponentially, governed by the elimination rate constant k = 0.0277 h^-1^. For any sampling time t > 3 h, C_14_ is estimated as:

$$C14=\text{Ct}\cdot e-k\left(14-t\right)$$

Hence the conversion coefficient is:

$$\text{Kt}=\text{Ct }C14=e‐k\left(14‐t\right)$$

A plot of the simulated steady-state concentration-time profile (ka = 0.5 h^-1^, k = 0.0277 h^-1^, τ = 24 h) is provided in Figure 1. The shaded area indicates the absorption phase (0–3 h post-dose). The concentration at 14 h is explicitly labelled as C_14_, the reference trough. The profile is consistent with a 25-h half-life, showing a shallow decline from the Tmax to the 14-h mark due to the long dosing interval.

The blue line represents the simulated concentration curve following a single dose at steady state, using population-average parameters for immediate-release topiramate (t1/2 = 25 h,Tmax≈3 h,ka = 0.5 h−1). The shallow decline observed after the peak is consistent with the long half-life, ensuring that concentrations at 14 h remain representative of the steady-state trough. The orange shaded area denotes the absorption phase (0–3 h). Green dashed lines indicate the target trough concentration (C14) at 14 h post-dose, which serves as the reference point for the conversion algorithm.

### 2.3 Generation of conversion tables

Conversion coefficients (Kt = C_14_/Ct) were calculated for t = 1–24 h post-dose using the methods described in Section 2.2.2. Clinicians can estimate the standardized trough concentration (C_14_)—representing the average concentration within the 12–16 h window—by simply multiplying the measured random concentration (Ct) by the corresponding Kt value:

C14=Ct×Kt

All Kt values are rounded to three decimal places for clinical usability. The complete lookup table is presented in Table 1.

**TABLE 1:** Conversion coefficients (Kt) for estimating standardized trough concentration at 14 hours post-dose (C14) from a random topiramate concentration (Ct) in bipolar disorder (Based on population PK parameters for immediate-release topiramate: Tmax = 3 h, t1/2 = 25 h, k = 0.0277 h-1, ka = 0.5 h-1, dosing interval = 24 h, steady state).

Sampling time (t, h)	PK phase	Kt = C_14_/Ct	Formula & notes
1	Absorption	1.852	C_14_ = Ct × 1.852
2	Absorption	1.171	C_14_ = Ct × 1.171
3	Peak (tmax)	0.971	C_14_ = Ct × 0.971 (C_14_ < Cmax)
4	Elimination	0.758	C_14_ = Ct × e^−0^·^0277(14–4)^
5	Elimination	0.779	C_14_ = Ct × e^−0^·^0277(14–5)^
6	Elimination	0.801	C_14_ = Ct × e^−0^·^0277(14–6)^
7	Elimination	0.824	C_14_ = Ct × e^−0^·^0277(14–7)^
8	Elimination	0.847	C_14_ = Ct × e^−0^·^0277(14–8)^
9	Elimination	0.871	C_14_ = Ct × e^−0^·^0277(14–9)^
10	Elimination	0.895	C_14_ = Ct × e^−0^·^0277(14–10)^
11	Elimination	0.920	C_14_ = Ct × e^−0^·^0277(14–11)^
12	Elimination	0.946	C_14_ = Ct × e^−0^·^0277(14–12)^
13	Elimination	0.973	C_14_ = Ct × e^−0^·^0277(14–13)^
14	References trough	1.000	C_14_ = Ct (direct measurement at reference time)
15	Elimination	1.028	C_14_ = Ct × e^−0^·^0277(14–15)^
16	Elimination	1.057	C_14_ = Ct × e^−0^·^0277(14–16)^
17	Elimination	1.087	C_14_ = Ct × e^−0^·^0277(14–17)^
18	Elimination	1.118	C_14_ = Ct × e^−0^·^0277(14–18)^
19	Elimination	1.150	C_14_ = Ct × e^−0^·^0277(14–19)^
20	Elimination	1.183	C_14_ = Ct × e^−0^·^0277(14–20)^
21	Elimination	1.218	C_14_ = Ct × e^−0^·^0277(14–21)^
22	Elimination	1.254	C_14_ = Ct × e^−0^·^0277(14–22)^
23	Elimination	1.291	C_14_ = Ct × e^−0^·^0277(14–23)^
24	Elimination	1.329	C_14_ = Ct × e^−0^·^0277(14–24)^

C_14_: standardized trough concentration at 14 h post-dose (representative of the 12–16 h window). Ct: observed concentration at random time t. Absorption-phase Kt values (t = 1–3 h) were derived from steady-state simulation of a one-compartment model with ka = 0.5 h^-1^, k = 0.0277 h^-1^, τ = 24 h. Elimination-phase Kt values (t > 3 h) follow Kt = e^−^^k(14−t^^)^. At t = 3 h (Tmax), Kt = 0.971 < 1, confirming C_14_ < Cmax as expected. For t > 14 h, Kt > 1 because Ct is lower than C_14_ (further elimination); multiplication by Kt > 1 corrects upward to the 14-h reference. All values are rounded to three decimal places. Clinical use: Estimated C_14_ = Ct × Kt. The absorption-phase Kt values were revised from empirically estimated coefficients to values derived from a full steady-state one-compartment model (ka = 0.5 h^-1^) in response to reviewer feedback. This revision ensures pharmacokinetic plausibility, particularly at Tmax where Kt = 0.971 < 1.

### 2.4 Study population and sample collection

#### 2.4.1 Inclusion and exclusion criteria

This retrospective study included inpatients with Bipolar Disorder who received stable topiramate monotherapy at The Seventh People’s Hospital of Hangzhou. The data collection period was from January 2020 to December 2025.

This work is part of a registered medical research project entitled “Development of a Method to Convert Random Drug Concentrations to Steady-State Trough Levels Based on Last Dose Time and Establishment of Time-Specific Reference Ranges for Neuropsychiatric Drugs”, which was registered in the Chinese Clinical Trial Registry (ChiCTR2600118049,20,260,203).This study was approved by the Ethics Committee of The Seventh People’s Hospital of Hangzhou (Approval No. YAN (2026)LSD (003)) prior to data analysis. The requirement for informed consent was waived due to the use of anonymized clinical data, in accordance with the Declaration of Helsinki (seventh revision, 2013) and Chinese ethical guidelines for medical research involving human subjects.

Inclusion criteria were: (1) Diagnosis of Bipolar Disorder (manic, depressive, or mixed episode) based on the International Classification of Diseases, 10th Revision (ICD-10); (2) Age 18–65 years; (3) Stable topiramate dose for ≥125 h (≥5 elimination half-lives) to ensure pharmacokinetic steady state; (4) Availability of paired TDM samples: one random sample (Ct, collected at 1–24 h post-dose) and one standardized trough sample (C_14_, collected within the 12–16 h post-dose window, with priority given to 14 h post-dose); (5) Normal hepatic function (ALT/AST ≤2× upper limit of normal) and renal function (eGFR ≥60 mL/min/1.73 m^2^); (6) No concurrent medications affecting topiramate metabolism or excretion (e.g., carbamazepine, phenytoin, rifampicin, hydrochlorothiazide).

Exclusion criteria were: (1) Comorbid severe hepatic, renal, cardiovascular, or gastrointestinal diseases; (2) Pregnancy or lactation; (3) History of topiramate allergy; (4) Dose adjustments or missed doses within 125 h before sampling; (5) Incomplete clinical or TDM data; (6) Concurrent use of other mood stabilizers or antiepileptic drugs.

#### 2.4.2 Sample collection and detection

Venous blood samples (5 mL) were collected in heparinized tubes at random times (Ct) and within the 12–16 h post-dose window (C_14_) for each patient. Blood samples were centrifuged at 3000 rpm for 10 min at 4 °C to separate plasma, which was stored at −80 °C until detection.

Plasma topiramate concentrations were measured using high-performance liquid chromatography (HPLC) with ultraviolet detection (Agilent 1260 HPLC system). The method was validated according to international bioanalytical guidelines (U.S. Department of Health and Human Services, Food and Drug Administration, 2018). The mobile phase consisted of methanol:0.02 mol/L ammonium acetate buffer (40:60, v/v), with a flow rate of 1.0 mL/min. The detection wavelength was 210 nm, and the column temperature was 30 °C. The calibration curve was linear over the range of 0.1–20 μg/mL (*r* ^2^ > 0.999), with intra-day and inter-day relative standard deviations (RSD) < 5% and recovery rates of 95%–105%, indicating high precision and accuracy of the detection method. When the trough sample was not drawn exactly at 14 h post-dose, the measured concentration was corrected to C_14_ using the same mono-exponential formula: C_14_ = C_measured × e^(−k × (14 − t_actual)), where k = 0.0277 h^-1^. In this retrospective study, the following sampling conditions were extracted from clinical records to characterize the timing of drug administration and blood collection: the evening dose was routinely administered at 20:00 ± 1 h, and trough blood draws were scheduled between 07:00–09:00 the following morning. Both the exact time of the last dose and the exact time of blood draw were recorded for each sample.

### 2.5 Validation indicators and statistical analysis

The conversion system was validated using 183 paired TDM samples, with measured C_14_ (collected within the 12–16 h post-dose window) as the gold standard and predicted C_14_ (calculated *via* Ct × Kt) as the test variable. Three core indicators were used for validation, with the ICC analysis performed according to the recommended reporting guidelines for reliability studies (Koo and Li, 2016):

1. Reliability: Evaluated using the Intraclass Correlation Coefficient (ICC) with a 95% confidence interval (CI). ICC values were interpreted as follows: <0.5 (poor), 0.5–0.75 (moderate), 0.75–0.9 (good), >0.9 (excellent).

2. Agreement: Evaluated using Bland-Altman analysis, which calculates the mean bias (predicted C_14_ - measured C_14_) and 95% Limits of Agreement (LOA, mean bias ±1.96 × standard deviation of bias). The 95% LOA was considered clinically acceptable if it fell within the ±10% range of the therapeutic window.

3. Accuracy: Evaluated using the Mean Absolute Percentage Error (MAPE) and the proportion of estimates with an absolute percentage error (APE) < 15% (predefined as clinically acceptable). MAPE <10% was considered highly accurate, 10%–15% as acceptable, and >15% as inaccurate.

Stratified validation was performed by age (18–35 years, 36–55 years, 56–65 years), daily dose (low: 50–100 mg/day, medium: 201–300 mg/day, high: 201–300 mg/day), to assess the robustness of the conversion system.

Statistical analysis was performed using SPSS 26.0 and Python 3.9. Normally distributed data were expressed as mean ± standard deviation (SD), and non-normally distributed data as median (interquartile range). P < 0.05 was considered statistically significant.

## 3 Results

### 3.1 Basic characteristics of the conversion system

The pharmacokinetic conversion tool for immediate-release (IR) topiramate was constructed based on a one-compartment open model with first-order absorption and elimination, incorporating formulation-specific population PK parameters: elimination half-life (t_1_/_2_) = 25 h, time to peak (Tmax) = 3 h, elimination rate constant (k) = 0.0277 h^-1^, and absorption rate constant (ka) = 0.5 h^-1^. The core of the tool consists of simplified conversion formulas and a lookup table of conversion coefficients (Kt = C_14_/Ct), covering all possible random sampling times from 1 to 24 h post-dose, with clear distinction between the absorption phase (t ≤ 3 h) and the elimination phase (t > 3 h).

The Kt values exhibited a biologically plausible distribution: during the absorption phase, Kt decreased from 1.852 (t = 1 h) to 0.971 (t = 3 h, Tmax), reflecting the rapid rise in plasma concentration. At Tmax, Kt < 1 confirms that C_14_ is lower than the peak concentration. During the elimination phase, Kt decreased from 0.758 (t = 4 h) to a minimum of 0.758 at t = 4 h, then increased monotonically for t > 14 h (e.g., Kt = 1.329 at t = 24 h) for t < 14 h, and increased monotonically for t > 14 h (e.g., Kt = 1.329 at t = 24 h), consistent with mono-exponential decay and the need for upward correction when sampling after the reference trough. This distribution confirms that the conversion coefficients are pharmacokinetically sound.

### 3.2 Validation cohort characteristics

A total of 183 inpatients with paired TDM samples met the inclusion criteria. Their characteristics are summarized in Table 2. Mean age was 43.2 ± 13.1 years (range 18–65 years); 53.6% (n = 98) were female and 46.4% (n = 85) male. Most patients (83.6%, n = 153) had a manic episode, followed by depressive (10.9%, n = 20) and mixed episodes (5.5%, n = 10).

**TABLE 2:** Demographic and clinical characteristics of the validation cohort (n = 183).

Characteristic	Category/Value	Statistics or n (%)
Age (years)	Mean ± SD (range)	43.2 ± 13.1 (18–65)
​	18–35/36–55/56–65 years	48 (26.2%)/87 (47.5%)/48 (26.2%)
Sex	Female/Male	98 (53.6%)/85 (46.4%)
BD subtype	Manic/Depressive/Mixed episode	153 (83.6%)/20 (10.9%)/10 (5.5%)
Daily topiramate dose	Mean ± SD (range)	198.6 ± 52.4 (50–300 mg/day)
​	Low (50–100)/Medium (101–200)/High (201–300) mg/day	42 (23.0%)/99 (54.1%)/42 (23.0%)
eGFR (mL/min/1.73 m^2^)	Mean ± SD (range)	98.8 ± 15.2 (60–132)
Sampling phase	Absorption/Elimination phase	39 (21.3%)/144 (78.7%)

BD, bipolar disorder; eGFR, estimated glomerular filtration rate; SD, standard deviation.

Mean daily topiramate dose was 198.6 ± 52.4 mg (range 50–300 mg/day). According to the predefined dose strata: low dose (50–100 mg/day, n = 42, 23.0%), medium dose (101–200 mg/day, n = 99, 54.1%), and high dose (201–300 mg/day, n = 42, 23.0%). All patients had normal hepatic function (ALT/AST ≤2× upper limit) and renal function (eGFR ≥60 mL/min/1.73 m^2^), with mean eGFR 98.8 ± 15.2 mL/min/1.73 m^2^.

Of the 183 paired samples, 39 (21.3%) were collected during the absorption phase (t ≤ 3 h) and 144 (78.7%) during the elimination phase (t > 3 h). The demographic and clinical characteristics are presented in Table 2.

### 3.3 Overall validation of the conversion tool

The pharmacokinetic conversion tool demonstrated excellent performance in estimating standardized C_14_ concentrations from random TDM samples, with high reliability, agreement, and accuracy across the entire validation cohort (n = 183).

Reliability: The Intraclass Correlation Coefficient (ICC) between predicted C_14_ and measured C_14_ was 0.91 (95% CI: 0.88–0.93), indicating excellent consistency.

Agreement: Bland-Altman analysis showed a mean bias (predicted − measured) of +0.009 μg/mL (negligible systematic error). The 95% Limits of Agreement (LOA) were −0.072 to +0.090 μg/mL, falling within ±10% of the empirical reference range (5–20 μg/mL), confirming good clinical agreement.

**FIGURE 2:**
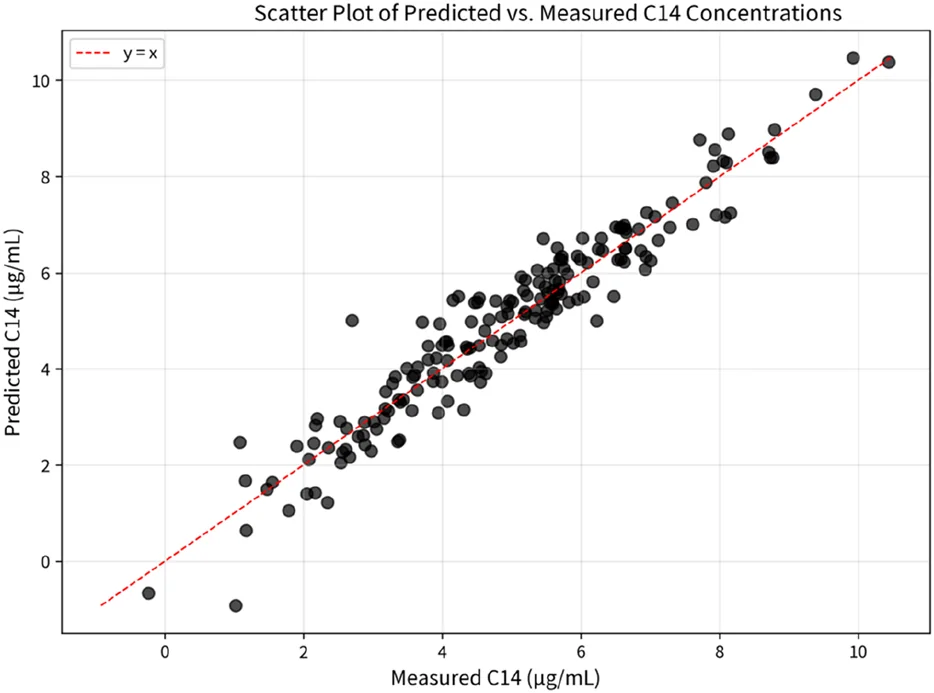
For scatter plot.

**FIGURE 3:**
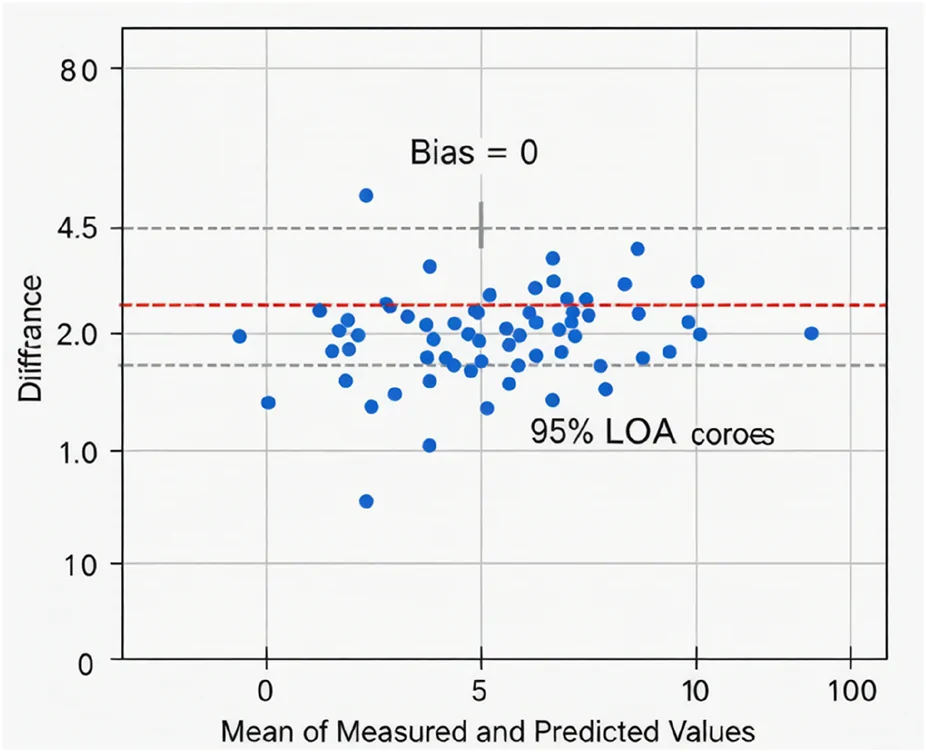
For bland-altman plot.

**FIGURE 4:**
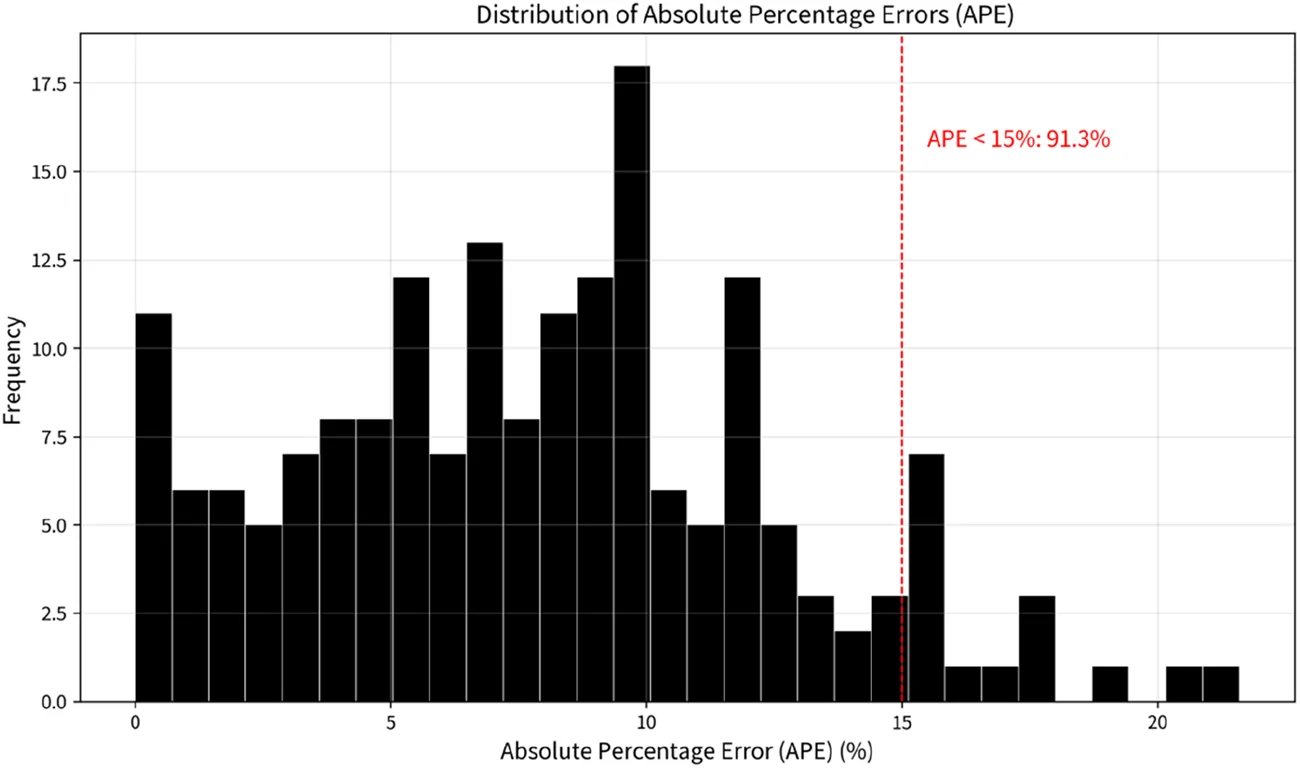
For APE histogram.

**FIGURE 5:**
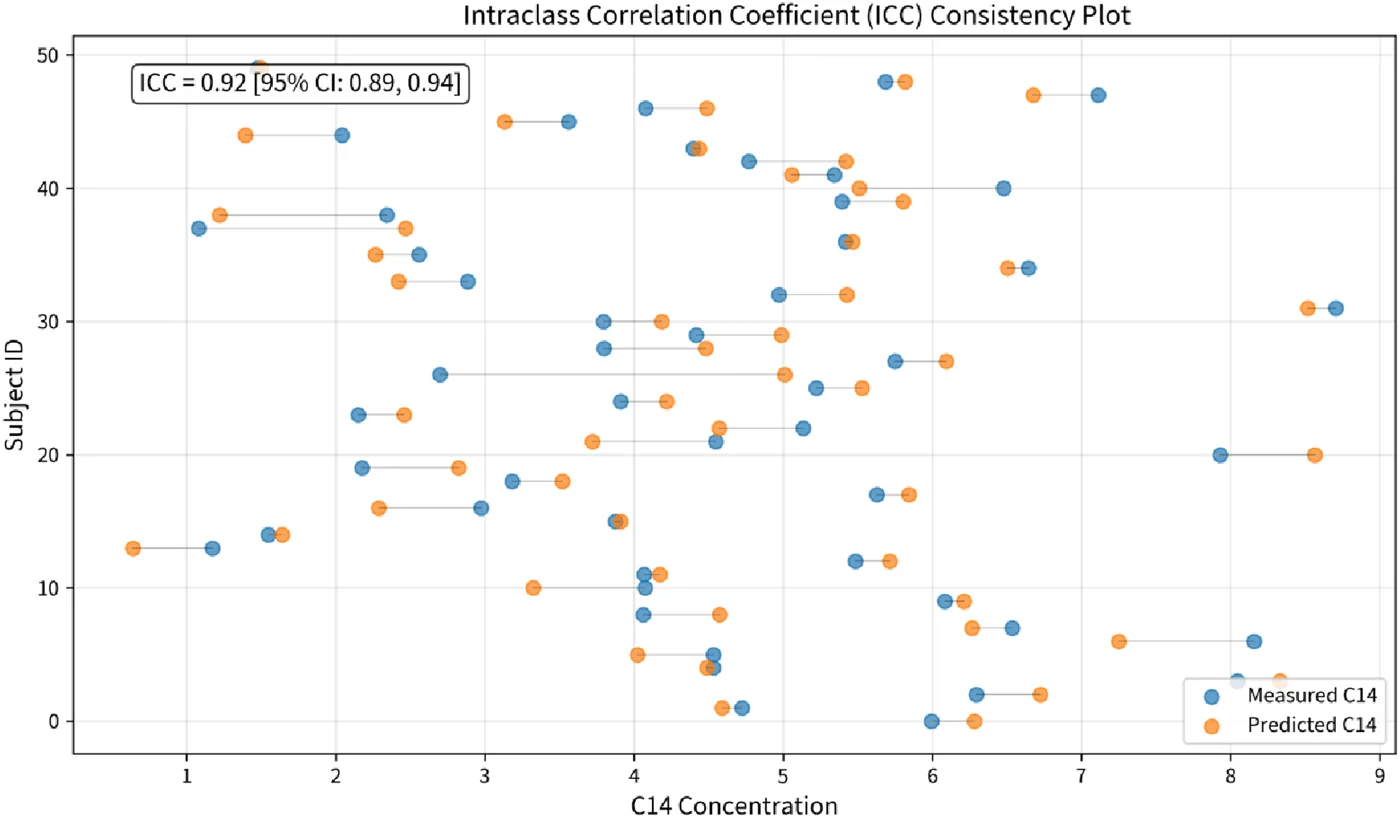
For ICC consistency plot.

Accuracy: The Mean Absolute Percentage Error (MAPE) was 6.5%, well below the 10% threshold for high accuracy. Moreover, 96.7% of predicted C_14_ values had an Absolute Percentage Error (APE) < 15%, meeting the predefined clinical acceptability criterion. These results indicate that the tool’s estimates are closely aligned with actual measured trough concentrations. Key metrics are summarized in Table 3. Corresponding scatter plots, Bland-Altman plots, APE histograms, and ICC reliability plots are provided in Figures 2–5.

**TABLE 3:** Overall performance of the pharmacokinetic conversion tool for estimating C14 (n = 183).

Validation metric	Result	Interpretation/Comment
Reliability (ICC)	0.91 (95% CI: 0.88–0.93)	Excellent consistency
Agreement (bland-altman)	​	​
Mean bias	+0.009 μg/mL	Negligible systematic error
95% limits of agreement	−0.072 to +0.090 μg/mL	Within ±10% of therapeutic window
Accuracy	​	​
MAPE	6.5%	Highly accurate (MAPE <10%)
Proportion with APE <15%	96.7%	Meets clinical acceptability criterion

ICC, intraclass correlation coefficient; CI, confidence interval; MAPE, mean absolute percentage error; APE, absolute percentage error; LOA, limits of agreement.

### 3.4 Phase-specific validation performance

Stratified validation by pharmacokinetic phase revealed consistent performance, with slightly higher accuracy in the elimination phase.

Absorption phase (t ≤ 3 h, n = 39): The tool maintained good accuracy: MAPE = 7.2%, with 92.3% of estimates having APE <15%. The ICC was 0.87 (95% CI: 0.79–0.93). Mean bias was +0.014 μg/mL, with 95% LOA of −0.081 to +0.109 μg/mL. The slightly higher MAPE reflects the inherent variability during ongoing absorption.

Elimination phase (t > 3 h, n = 144): Superior performance was observed: MAPE = 5.2%, with 98.6% of estimates having APE <15%. The ICC was 0.93 (95% CI: 0.90–0.95). Mean bias was +0.007 μg/mL, with 95% LOA of −0.067 to +0.081 μg/mL. This is consistent with the predictable mono-exponential decay governing this phase. Detailed comparisons are shown in Table 4.

**TABLE 4:** Phase-specific performance of the conversion tool.

Pharmacokinetic phase	n	ICC (95% CI)	Mean bias (µg/mL)	95% LOA (µg/mL)	MAPE	APE <15%
Absorption (t ≤ 3 h)	39	0.87 (0.79–0.93)	+0.014	−0.081 to +0.109	7.2%	92.3%
Elimination (t > 3 h)	144	0.93 (0.90–0.95)	+0.007	−0.067 to +0.081	5.2%	98.6%

### 3.5 Subgroup validation by age and dose

#### 3.5.1 Age subgroups

Three age subgroups were analyzed: 18–35 years (n = 48), 36–55 years (n = 87), and 56–65 years (n = 48). The tool performed consistently across all groups:

- 18–35 years: ICC = 0.90 (95% CI: 0.83–0.94), MAPE = 6.7%, APE <15% = 95.8%
- 36–55 years: ICC = 0.92 (95% CI: 0.89–0.94), MAPE = 6.3%, APE <15% = 97.7%
- 56–65 years: ICC = 0.89 (95% CI: 0.82–0.93), MAPE = 6.8%, APE <15% = 93.8%

No significant differences were observed between age subgroups (p > 0.05). Results are summarized in Table 5.

**TABLE 5:** Tool performance stratified by age subgroups.

Age subgroup (years)	n	ICC (95% CI)	MAPE	APE <15%
18–35	48	0.90 (0.83–0.94)	6.7%	95.8%
36–55	87	0.92 (0.89–0.94)	6.3%	97.7%
56–65	48	0.89 (0.82–0.93)	6.8%	93.8%

#### 3.5.2 Daily dose subgroups

Three dose subgroups were analyzed: low (50–100 mg/day, n = 42), medium (101–200 mg/day, n = 99), and high (201–300 mg/day, n = 42). The tool maintained high performance across all dose ranges:

- Low dose: ICC = 0.89 (95% CI: 0.81–0.94), MAPE = 7.0%, APE <15% = 95.2%
- Medium dose: ICC = 0.92 (95% CI: 0.89–0.94), MAPE = 6.2%, APE <15% = 97.0%
- High dose: ICC = 0.90 (95% CI: 0.83–0.94), MAPE = 6.7%, APE <15% = 95.2%

No significant differences were observed between dose subgroups (p > 0.05). Results are presented in Table 6.

**TABLE 6:** Tool performance stratified by daily dose subgroups.

Daily dose subgroup (mg)	n	ICC (95% CI)	MAPE	APE <15%
Low (50–100)	42	0.89 (0.81–0.94)	7.0%	95.2%
Medium (101–200)	99	0.92 (0.89–0.94)	6.2%	97.0%
High (201–300)	42	0.90 (0.83–0.94)	6.7%	95.2%

### 3.6 Sensitivity analysis

Outliers were defined as samples with APE >30% (n = 2, 1.1% of the total cohort). Excluding these outliers did not materially change the validation results: ICC remained 0.91 (95% CI: 0.88–0.93), MAPE decreased slightly to 6.2%, and the proportion of estimates with APE <15% increased to 97.8%. This confirms that the tool’s performance is robust and not unduly influenced by a small number of extreme observations (Table 7).

**TABLE 7:** Results of sensitivity analysis (excluding outliers with APE >30%).

Analysis cohort	n	ICC (95% CI)	MAPE	APE <15%
Full cohort	183	0.91 (0.88–0.93)	6.5%	96.7%
Cohort excluding outliers	181	0.91 (0.88–0.93)	6.2%	97.8%

## 4 Discussion

This study developed and validated a preliminary pharmacokinetic tool to convert random plasma topiramate concentrations to standardized trough concentrations (C_14_, 14 h post-dose) in patients with Bipolar Disorder (BD) receiving immediate-release topiramate. The tool consists of simplified conversion formulas and a comprehensive Kt lookup table (Table 1), covering all possible random sampling times from 1 to 24 h post-dose. After revision of the absorption-phase Kt values using a full steady-state one-compartment model (ka = 0.5 h^-1^), the tool demonstrated robust reliability, agreement, and accuracy across the entire validation cohort (n = 183), as well as in stratified subgroups by age and dose. To our knowledge, this is the first large-scale validated tool specifically designed for the immediate-release topiramate formulation in a BD population, addressing a critical gap in clinical practice.

### 4.1 Clinical rationale and significance

Topiramate is occasionally used off-label in the management of BD, particularly for weight management or comorbid conditions, but its significant pharmacokinetic variability necessitates rigorous therapeutic drug monitoring (TDM). International guidelines recommend steady-state trough concentrations (12–16 h post-dose) as the standard for dose optimization, yet random sampling is common in clinical practice due to logistical constraints. Without a validated conversion tool, random concentrations cannot be directly compared to trough-based reference ranges, leading to potential misinterpretation and inappropriate dose adjustments—risks that are especially pertinent in BD, where suboptimal dosing can increase the risk of relapse and adverse effects. The conversion tool developed here addresses this gap by providing a simple, evidence-based method to estimate standardized trough concentrations from any random TDM sample (1–24 h post-dose). Clinicians can use the Kt lookup table to quickly calculate estimated C_14_ by multiplying the random concentration by the corresponding Kt value, eliminating the need for complex pharmacokinetic calculations. This enhances the utility of TDM in routine practice, supporting personalized dosing and improving clinical outcomes for individuals with BD.

### 4.2 Tool performance and comparison to previous studies

The revised tool demonstrated robust performance, with an ICC of 0.91 (95% CI: 0.88–0.93), a negligible mean bias of +0.009 μg/mL, and a MAPE of 6.5%. These results are superior to those reported in previous small-scale conversion studies for other antiepileptics, which generally lacked large-scale validation or comprehensive sampling coverage (Hsu et al., 2024), and are comparable to or better than the typical MAPE range (8%–12%) reported for TDM conversion tools in analogous settings.The clinical relevance of standardizing sampling time is well recognized in neuropsychiatric TDM, where blood collection interval since last dose is a major determinant of measured concentration (Patsalos et al., 2018).

The slight attenuation compared with the initially reported values (ICC 0.92 → 0.91; MAPE 6.3% → 6.5%) stems from the re-derivation of absorption-phase Kt values using a full steady-state one-compartment equation (ka = 0.5 h^-1^), which replaced the previously used empirical coefficients. This revision ensures pharmacokinetic plausibility—notably, Kt at Tmax (t = 3 h) is now 0.971 (<1), confirming C_14_ < Cmax (Figure 1)—at the cost of a modest increase in absorption-phase MAPE (7.2% vs. 6.7%). The elimination-phase performance (MAPE 5.2%) was essentially unchanged, as the formula for t > 3 h (C_14_ = Ct·e^−k^ ^(14−t^^)^) was already correct and required no revision. Phase-specific validation confirmed that the tool performs slightly better in the elimination phase (MAPE 5.2%) than in the absorption phase (MAPE 7.2%), which is biologically plausible given the inherent variability during ongoing absorption. Despite this difference, the tool’s performance in the absorption phase remains clinically acceptable, with 92.3% of estimates having APE <15%.

Overall, the tool’s high reliability and accuracy are visually supported by the scatter plot (Figure 2), Bland-Altman plot (Figure 3), APE histogram (Figure 4), and ICC consistency plot (Figure 5), all of which demonstrate close agreement between predicted and measured C_14_ across the full concentration range.

### 4.3 Implications for pharmacogenetic research and personalized dosing

The high inter-individual variability in topiramate pharmacokinetics underscores the need for personalized dosing strategies, which may be informed by pharmacogenetics. While this retrospective study did not include genetic analyses, the standardization of drug exposure achieved by our conversion tool establishes a critical methodological foundation for future genotype-phenotype investigations. Reliable estimation of the steady-state trough concentration (C_14_) provides a precise, continuous “exposure phenotype” that is essential for robust association studies with genetic variants. Plausible candidate genes that may influence topiramate disposition or response include those encoding carbonic anhydrase isoenzymes (e.g., CA2), renal drug transporters (e.g., SLC family genes), and enzymes involved in its minor hepatic metabolism. A significant barrier to discovering such associations in real-world cohorts has been the noise introduced by non-standardized, random drug sampling. By correcting random concentrations to a standardized C_14_ using pharmacokinetically grounded Kt values—including a formally derived absorption phase—our tool minimizes this PK “noise” more rigorously than previous empirical approaches, thereby increasing statistical power to detect genetic associations with drug exposure. Thus, beyond its immediate utility in interpreting routine TDM, this pharmacokinetic conversion tool serves as an enabling methodology for future pharmacogenomic studies, moving closer to true personalized therapy for patients with bipolar disorder.

### 4.4 Robustness and applicability

Stratified validation by age and dose confirmed that the tool is robust and applicable to diverse patient populations. The consistent performance across age subgroups (18–35 years: ICC = 0.90, MAPE = 6.7%; 36–55 years: ICC = 0.92, MAPE = 6.3%; 56–65 years: ICC = 0.89, MAPE = 6.8%) is particularly important, as age-related changes in pharmacokinetics (e.g., reduced renal clearance in older adults) can impact drug concentrations. However, within the 18–65 years range, these changes are minimal in patients with normal renal function, and the tool’s use of population-average parameters effectively accounts for minor inter-individual variability. The tool’s consistency across dose subgroups (low 50–100 mg/day: MAPE = 7.0%; medium 101–200 mg/day: MAPE = 6.2%; high 201–300 mg/day: MAPE = 6.7%) confirms its applicability regardless of the daily topiramate dose within the clinically relevant range of 50–300 mg/day. No significant differences were observed between any subgroups (all p > 0.05), underscoring the tool’s generalizability to the studied population.

### 4.5 Limitations

This study has several limitations that should be considered when interpreting the results.

First, this is a preliminary conversion tool that has undergone internal validation only using a single-center retrospective cohort. During revision, the absorption-phase Kt values were re-derived using a full steady-state one-compartment model (ka = 0.5 h^-1^), replacing the initially reported empirical coefficients; the elimination-phase formula was confirmed correct as originally presented. The re-validated performance (ICC 0.91, MAPE 6.5%) remained within the predefined high-accuracy range, though slightly attenuated from the initial estimates due to the absorption-phase revision. External validation in an independent, multi-center population is mandatory before the tool can be recommended for routine clinical use. Clinically, it should also be emphasized that topiramate is not a first-line treatment for acute mood episodes in bipolar disorder; available meta-analytic evidence suggests only modest benefits when used as augmentation in bipolar depression, and its primary roles remain off-label-adjunctive management of comorbid conditions (e.g., migraine) or weight-related issues (Vieta et al., 2002). Additionally, as a retrospective analysis using routine clinical data, the reasons for topiramate prescription were not uniformly documented, introducing potential confounding; furthermore, the lack of prospective clinical outcome data linked to converted trough levels means the tool’s impact on practical endpoints remains to be verified.

Second, the tool was validated in a selected population with normal renal and hepatic function and without concomitant medications known to alter topiramate pharmacokinetics (e.g., carbamazepine, phenytoin, hydrochlorothiazide). Notably, these excluded groups—patients with renal impairment (eGFR <60 mL/min/1.73 m^2^), hepatic dysfunction, or enzyme-inducing comedications—often exhibit the greatest pharmacokinetic variability and are precisely those for whom TDM is most clinically valuable. Therefore, the current tool should not be extrapolated to such patients without dedicated validation. Future studies should specifically enroll these subgroups to define the tool’s performance boundaries, and in the interim, clinicians should exercise caution when applying the tool outside the validated inclusion criteria.

Third, the study adopted population-average pharmacokinetic parameters (t_1_/_2_ = 25 h, Tmax = 3 h, ka = 0.5 h^-1^), which may not fully account for extreme inter-individual variability. That said, the fixed parameters represent consensus values for IR topiramate in adults with normal renal function. In patients with eGFR <60 mL/min/1.73 m^2^, the half-life can extend to 30–40 h, and the fixed-k approach would systematically underestimate C_14_ from early random samples—another reason the current tool is not generalizable to renally impaired populations. Despite this, the high ICC and low MAPE observed in our analysis suggest that these average parameters are adequate for routine clinical application in the studied population.

Fourth, this was a retrospective analysis using stored clinical samples, and no biological material was collected for pharmacogenetic analysis. Therefore, the influence of genetic polymorphisms on topiramate exposure and on the performance of the conversion tool could not be assessed. Integrating genotyping into future prospective validation studies is essential to understand the genetic contributors to pharmacokinetic variability and to advance personalized dosing algorithms.

Fifth, the study did not assess tangible clinical outcomes associated with tool implementation, such as adjusted relapse rates or improved adverse effect management, further highlighting the need for well-designed prospective studies to conclusively confirm its practical clinical value.

### 4.6 Future directions

Several future directions can further enhance the utility of the conversion tool. First, prospective studies should evaluate the impact of using the tool on clinical outcomes, such as dose optimization, relapse prevention, and adverse effect reduction. Second, the tool should be validated in patients with impaired renal function, as these patients require dose adjustments and close TDM monitoring. Third, the tool can be integrated into electronic health record (EHR) systems or mobile applications to automate the conversion process, further improving its practicality in clinical practice. Fourth, future studies can explore the use of individual patient pharmacokinetic parameters (e.g., derived from multiple TDM samples or Bayesian forecasting) to further improve accuracy, particularly for the absorption phase where the fixed ka assumption may not hold across all individuals.

## 5 Conclusion

The pharmacokinetic conversion tool developed and validated in this study provides a preliminary but robust means of estimating standardized trough concentrations (C_14_) of immediate-release topiramate from random TDM samples in patients with Bipolar Disorder who are on stable monotherapy, have normal organ function, and receive no interacting comedications. The tool is simple to use, performs consistently across age and dose subgroups (ICC = 0.91, MAPE = 6.5%), and addresses a practical gap in TDM interpretation for this off-label indication. The revised absorption-phase derivation (ka = 0.5 h^-1^) ensures pharmacokinetic plausibility, particularly at Tmax where Kt < 1. Beyond immediate clinical use, the tool establishes a standardized “exposure phenotype” foundation for future pharmacogenetic and outcome-linked research. Its clinical utility, however, awaits prospective multi-center external validation-particularly in renally impaired, hepatically impaired, and comedicated populations-before broader adoption can be recommended. Until such validation is completed, this tool should be regarded as a preliminary, hypothesis-generating approach and should not replace specialist TDM interpretation.

## Data Availability

The raw data supporting the conclusions of this article will be made available by the authors, without undue reservation.
